# Sleeping position and reported night-time asthma symptoms and medication

**DOI:** 10.11604/pamj.2016.24.59.9159

**Published:** 2016-05-13

**Authors:** Admirabilis Beno Kalolella

**Affiliations:** 1 Ifakara Health Institute, Dar es Salaam, Tanzania

**Keywords:** Nighttime asthma symptoms, nighttime sleep diary, bronchial asthma

## Abstract

A 49 years old man, known case of bronchial asthma for 43 years, with history of frequent asthmatic attacks, usually responding to double dose of intravenous Aminophylline and double dose of Hydrocortisone was received at medical emergency care unit at midnight with night-time asthma attack. The attack did not settle with Aminophylline single Intravenous injection. He was then admitted and put in supine sleep position for re-evaluation while his asthma symptoms were monitored while waiting for the medical officer's evaluation of his asthma status. After 3 hours of observation, asthma symptoms were relieved, and patient was discharged home and advised to sleep in supine position throughout every night to prevent asthma symptoms. The patient was followed up through nighttime sleep diary for one month. After one month period of monitoring, the patient had significance reduction in asthma symptoms and reduced night time medication, reduced episodes of night awakening due to asthma symptoms, and improved capability for normal works. This case report describes a novel approach of management and prophylaxis of asthmatic episodes through sleeping position that reduces and control asthma symptoms resulting in reduced drug consumption.

## Introduction

Bronchial Asthma is a syndrome condition or group of symptoms that are characterized by reversible airway obstruction as a result of hyper reactivity of the airways passage to allergens [1]. The hyper reaction may result into obstruction of airflow that presents asthma symptoms i.e. Wheezing, difficulty in breathing, coughing, chest tightness, chest pain, weight on chest, shortness of breath and mucus [1]. These symptoms can either settle by themselves or resolved through treatment with bronchodilators and corticosteroid [1, 2]. Night time bronchial asthmatic attack is very common in poorly controlled asthma [2]. Night time asthma symptoms are very distressing for patients because the condition awakens the patient, necessitating the patient to take drugs at night and limiting patient activity the following day [1, 2]. About 61% of asthma patients report nighttime asthma symptoms [2]. Adult asthmatics experience asthma related difficulties almost 3 to 4 times per week, with bothersome symptoms which include wheezing, difficulty in breathing, coughing, chest tightness, chest pain, weight on chest, shortness of breath, mucus and feelings of being tiredness that limit capability to have normal activity the following days after nighttime symptoms that often result in absenteeism at work [1, 2].

Studies on sleep and sleep position have been associated to have an effect on physical health [3]. Some studies indicate that sleeping positions have been linked with patho physiology state of the body [4]. Sleep position has also been associated with normalization of health conditions [5]. Increasing cases of nighttime asthma symptoms are common due to poor control of disease, and are seen at peripheral community health care facilities that distress nightshift staffs due lack of disease management skills and unavailability of emergency drugs to relieve the attack [2]. This case study report is of a patient among many cases of night time asthmatics who had been receiving inadequate treatment for persistent asthmatic attack, which is very common in our many health care centers that have persistent shortage of staffs with adequate knowledge and experience in treating acute attacks of asthma. The successful asthmatic symptoms reduction through sleeping position documented is presented here for the purpose of a case report. By providing this information on the success of the use of a sleep position will be resourceful for asthmatic patients, parents with asthmatic children, care takers and clinicians on the availability of a very effective home based measure that can easily be practiced by any asthma patient at home.

## Patient and observation

A 49-years- old man was received at midnight at a community health centre with night-time acute attack of bronchial asthma. On arrival at the medical centre, he was complaining of persistent cough with mucus, difficulty in breathing and shortness of breath, chest tightness, and chest pain with weight on chest. The history indicated that the patient experienced daily nighttime asthmatic attacks that always awakened him to take relieving drugs. He took Ketotifen tablets 1 mg at night, tablets Salbutamol 10mg three times a day and tablets Prednisolone 10mg once a day as treatment and prophylactic therapy on a regular basis.

The patient also used Salbutamol inhaler not less than 3 times per day during episodes of difficulties in breathing. Three days earlier he had been seen at the centre reporting with severe attack of bronchial asthma, and was treated with intravenous Aminophylline 250mg bolus, supported with equal dose given in 5% Dextrose 500mls drip solution to run slowly for 2 hrs, and intravenous Hydrocortisone 200mg that settled his acute attack. During chest examination, the patient experienced severe difficulty in breathing. Chest auscultation revealed crepitating and wheezing sounds indicating a severe acute attack of asthma.

### Treatment/method

The clinician managed the patient with intravenous Aminophylline 250mg that did not settle the acute attack. The patient was observed for 30 minutes and recorded a worsening of the asthma symptoms. Due to this worsening of the presenting symptoms, the patient was admitted and laid on a hospital functional bed in a supine sleeping position while his asthma symptoms monitored, awaiting re - evaluation of his asthma status.

The clinician monitoring the asthmatic symptoms observed that there was a reduced struggle of breathing 30 minutes after laying the patient in the supine sleep position. The clinician continued to record asthma symptoms for 3 more hours to document the progress before the patient was re- evaluated by the medical officer for further management (Table 1).

**Table 1:** Asthma symptoms before and after supine sleep position

Asthma Symptoms	Time interval of Asthma symptoms observation in minutes							
	Before	After						
	0	30	60	90	120	150	180	remarks
Chest Tightness	√	√						
Chest Pain	√	√	√	√	√	√	√	
Pressure/Weight on Chest	√	√	√					
Difficulty Breathings	√							
Wheezing	√	√	√					
Shortness of Breath	√							
Cough	√	√		√			√√	
Mucus	√	√						
***Reduction of Asthma Symptoms (shaded cells)***	***0/8***	***2/8***	***5/8***	***6/8***	***7/8***	***7/8***	***7/8***	

### Outcome and follow-up

The patient was discharged having improved from his asthmatic attack status without any additional medicine and advised to sleep in supine position throughout every night to prevent asthma symptoms. The patient was followed up through nighttime sleep diary for one month to monitor nighttime asthma symptoms, night awakening episodes due to asthma symptoms, and activity limitation [6–8].

Nighttime sleep diary is easy to use instrument to monitor daily asthma symptoms experienced at night that are characterized by awakening at night cause by asthma symptoms such as coughing, tightness of chest, shortness of breath at night experienced at night and filled up the morning after waking up, requiring the use of reliever medication at night which sometimes do not work to treat these symptoms and tiredness (Table 2) [7, 8]. The patient was instructed to report to the health centre every Monday for re evaluation of his asthma condition. The diary table presented all symptoms and medication that the clinician could see and scrutinize troublesome persistence symptoms, relief medication taken and night awakening episodes and be able to calculate percentage of reduction of the same [7, 8].

**Table 2:** Nighttime sleep diary for asthma symptoms and medication

	Day of Week: Date…………………………………Complete in the morning							
Symptoms	Day 1	Day 2	Day 3	Day 4	Day 5	Day 6	Day 7	Remarks
Chest Tightness								
Chest Pain	√						√	
Pressure/Weight on Chest	√	√						
Difficulty Breathing								
Wheezing								
Shortness of Breath								
Cough	√	√	√			√		
Mucus	√	√						
***Reduction of nighttime Asthma symptoms (shaded cells)***	***4/8***	***5/8***	***7/8***	***8/8***	***8/8***	***7/8***	***7/8***	
Night Awakenings due to asthma symptoms	√	√						
Activity limitation	√							
**Medication taken at night**	**Day 1**	**Day 2**	**Day 3**	**Day 4**	**Day 5**	**Day 6**	**Day 7**	
Salbutamol tablets	√							
Prednisolone tablets	√							
Ketotifen tablets								
Serotide inhaler (Salmeterol + fluticasone)								
Salbutamol inhaler	√	√						
***Reduction of nighttime medication (shaded cells)***	***2/5***	***4/5***	***5/5***	***5/5***	***5/5***	***5/5***	***5/5***	

After one month period of monitoring, the patient had significant reduction in night time asthma symptoms, reduced medication, one or none night awakening episodes and no limitation of normal activities (Table 2). This case report describes a novel approach of management of uncontrolled asthma that usually result in nighttime asthma symptoms through the poisoning of the patient in a sleeping supine position at night.

## Discussion

During observations at the health centre, the reduction in asthma symptoms started just 30 minutes after supine sleep position that relieved difficulties in breathing and shortness of breath. After one hour, the patient had additional improvement with relieved chest tightness and coughing. At about 90 minutes of observation, the patient had stabilized with only the cough remaining as an asthma symptom. All asthma symptoms, except chest pain, were settled, during the observation of the remaining one and half hour (Table 1).

During the patient's one month follows up schedule the following was recorded: first week, day 1, the patient reported relief from chest pain, chest tightness, difficulty in breathing, shortness of breath and wheezing. However he was awakened at night due to chest pain, weight on chest and cough for which he took and took Salbutamol tablets, Salbutamol inhaler and Prednisolone tablets, and reported mild activity limitation; on day 2 similar improvements were reported, this time with additional improvement of chest pain. The patient was awakened at night due to weight on chest and cough for which he took medications of Salbutamol inhaler only. No activity limitation was reported; on day 3, the patient experienced only cough at night but did not wake up to take any medicine, and no activity limitation was reported; day 4, 5 were uneventful; day 6 and day 7 the patient experienced a single symptom cough and chest pain that did not awaken him for medication, and no activity limitation was reported; the report for week 2 and 3 were uneventful; however, the patient reported one day during week four that he had experienced chest tightness that awakened him to use the Salbutamol inhaler, but no activity limitation observed (Table 2). The patient reported struggle and difficulties to adapt to supine sleep position that can be reduced by Sleep Positioning Device.

The follow up cards of self reporting which were brought to the centre every week indicated considerable change with respect to reduction of night time asthma symptoms, reduction of night time asthma medication, reduced night awakening episodes due asthma symptoms, and reduced activity limitation. This case report shows the benefit of supine sleep position as measure to prevent and treat nighttime asthma symptoms. This patient did not report asthma attack for the whole period of follow up of one month.

## Conclusion

Supine sleep position for asthma patient has demonstrated that persistent use of this natural body position when sleeping ensures high reduction of asthma symptoms and bothersome night awakening episodes, consumption of ant-asthma medicine and reduces work limitation for patient. Supine sleep position can easily be used and adopted by any asthma patient as treatment and prophylactic measure against asthma symptoms without additional resources. Future research on supine sleep position should focus on construction and evaluation of efficacy of supine sleep position device for large scale distribution.
